# Infertility clinics and acupuncture: a qualitative web-based study

**DOI:** 10.1007/s10815-023-02898-6

**Published:** 2023-08-01

**Authors:** Bethany Magee, Kevin Richard Smith

**Affiliations:** Division of Health Science, School of Applied Sciences, Abertay University, Dundee, DD1 1HG UK

**Keywords:** Acupuncture, Complementary and alternative medicine (CAM), Infertility, Medical ethics

## Abstract

The increasing demand for fertility treatments has led to the rise of private clinics offering complementary and alternative medicine (CAM) treatments. The most frequently offered CAM infertility treatment is acupuncture. However, there is no good evidence to support the effectiveness of acupuncture in treating infertility. This study evaluates the scope of information provided by CAM fertility clinics in the UK. A content analysis was conducted on 200 websites of CAM fertility clinics in the UK that offer acupuncture as a treatment for infertility. Of the 48 clinics that met the eligibility criteria, the majority of the websites did not provide sufficient information on the efficacy, risks and success rates of acupuncture for infertility. This has the potential to infringe on patient autonomy, provide false hope and reduce the chances of pregnancy ever being achieved as fertility declines during the time course of ineffective acupuncture treatment.

**Supplementary information:**

The online version contains supplementary material available at 10.1007/s10815-023-02898-6.

## Introduction

In the UK and other western nations, for social and financial reasons, many would-be parents are increasingly delaying conception [1–3]. Because fertility (especially for women) declines with age, this demographic shift means that more people are running into difficulties in achieving and maintaining pregnancy [3, 4]. At the same time, the development of assisted reproductive technology (ART) has increasingly offered medical solutions to infertility—albeit at a price, both financial and psychological.

The demand for services that enable or enhance fertility is concomitantly high, and this creates an incentive for the commercial provision of services marketed at individuals who are experiencing fertility problems. Most of the services offered by *bona fide* ART clinics—those regulated by the Human Fertilisation & Embryology Authority (HFEA)—are based on scientifically proven ART approaches, such as in vitro fertilisation (IVF) and intra-cytoplasmic sperm injection (ICSI).^1^

By contrast, fertility treatments that are scientifically implausible and lack convincing evidence of effectiveness may be offered by private fertility clinics specialising in ‘complementary and alternative medicine’ (CAM). Examples of the modalities offered include acupuncture, fertility massage, herbal medicine, homeopathy, hypnotherapy and reflexology. Informal web searching has shown the existence of several CAM clinics offering fertility treatments in the UK, with acupuncture being by far the most common form of CAM promoted—with over three quarters of clinics offering it [5].

Acupuncture entails the insertion of needles into specific areas of the body which are claimed to correspond to internal organs, with a view of generating positive therapeutic effects. Traditional acupuncture is based on implausible physiological features and mechanisms, including ‘Qi’ (life energy) flowing through ‘meridians’ (body channels). No such ‘energy’ has ever been detected—despite decades of advanced scientific research into physiological communication and signalling systems. Nor have any such ‘channels’ been revealed, despite decades of painstaking research into the anatomy and physiology of human tissues and organs.

Some contemporary acupuncturists claim that acupuncture can treat disease by inducing neurophysiological, hormonal or immunological changes through the stimulation of sensory nerves in the skin and muscles [6–8]. In the context of infertility, various claims have been made that acupuncture can promote ovulation or enhance sperm production, by improving blood flow to the reproductive organs, regulating levels of reproductive hormones or reducing stress [9–12].

However, direct evidence in support of the existence of such mechanisms is limited. Moreover, there exists insufficient credible evidence from clinical trials to substantiate the ability of acupuncture to treat infertility beyond placebo effectiveness. Claimed evidence of the effectiveness of acupuncture has been published in academic journals. However, the overall publication landscape (despite the omnipresent phenomenon of publication bias resulting in an excess of ‘positive’ reports) paints a negative picture of the effectiveness of acupuncture treatments, with individual positive reports most plausibly viewed as outliers, i.e. statistical ‘false positive’ trial outcomes, or studies that are otherwise flawed in design, execution or interpretation [13].

The promotion and selling of acupuncture as a potential treatment for infertility raises substantive ethical issues. The longer the individual delays accessing effective treatment, the less likely it is that a successful pregnancy will (ever) ensue. So, while a patient using acupuncture for a non-serious condition (such as recurrent headaches) will benefit fully whenever they choose to switch to conventional medical treatment, a woman suffering from infertility will lose precious biological time the longer that acupuncture is used instead of established assisted conception treatment.^2^ A real danger for such a patient is that a potentially remediable case of infertility may become intractable through time wasted with acupuncture treatment.

The extent to which acupuncture may be offered and promoted by CAM fertility clinics in the UK has not been systematically explored to date. To address this important question, we conducted a web-based study of CAM fertility clinics in the UK in 2022, in order to evaluate the scope for patients being misled by the information provided to them on the CAM fertility clinics’ websites.

## Methods

### Design

A web-based content analysis was conducted for this study [14]. The approach used by a prior study was adapted for this purpose [15]. Google was used as a search engine, and a filter was applied to limit search results to pages from the UK. The search keyword used was ‘fertility CAM acupuncture clinic’. The search results were screened to find the first 200 websites that were relevant, i.e. CAM fertility clinics offering acupuncture related to fertility. (These websites were all present within the first 20 pages of search results). The data were collected in March 2022, thus giving a snapshot of CAM fertility clinics at that time. The eligibility and website selection criteria are detailed below.

### Eligibility

Websites were included if they met the following conditions: (1) acupuncture is offered as an infertility treatment; (2) the CAM fertility clinic has two or more acupuncturists; (3) the acupuncture is delivered as an in-clinic service; and (4) the clinic is located in the UK.

### Website selection

For final inclusion, the entire content of all 200 websites was screened, and it was determined whether the eligibility criteria were met. Clinics which were not solely focused on fertility-based treatments were not excluded as long as they had a page on their website with information regarding acupuncture for fertility, or therapists who specialised in fertility acupuncture. Websites that represented the same clinic but appeared multiple times were only analysed once.

### Variables

Ten pre-defined variables were used to characterise the clinics. The variables sought to define the information they provide in a standardised way so CAM fertility clinics can be easily compared. Data were collected for the categories shown in Table 1.

**Table 1:** Variables used in the analysis of UK-based CAM fertility clinic websites

Category	Website content
Acupuncture basics	An explanation of what acupuncture is
Procedure	A description of how the acupuncture will be carried out
Fertility claims	An explanation of how acupuncture may help with infertility
Success rates	Data are presented that indicate the clinic’s success rate in terms of fertility restoration
Disclaimer	The website makes it clear that acupuncture may not work for infertility
Risks	The risks of acupuncture are made clear
Side effects	The possible side effects of acupuncture are made clear
Patient reviews	Reviews from previous patients of the clinic are presented
Education/training	Details are provided of the level of training and education of the clinic’s acupuncturists
Timings and duration	An indication is given as to when the treatment should be started and for how long it should be continued

### Scoring system

All identified CAM fertility clinics meeting the inclusion criteria were assigned a score to determine whether there is likely potential for prospective patients without medical or scientific knowledge to receive misleading information. Table 2 sets out the scoring system used to determine a clinic information score (CIS) for various criteria for the CAM fertility clinics.

**Table 2:** Scoring of variables

Query criterion	Points allocation
Does the website give a clear and valid explanation of what acupuncture is?	1 Point if yes, 0 points if no
Does the website describe in sufficient detail how the acupuncture will be carried out?	1 Point if yes, 0 points if no
Does the website offer a clear explanation, understandable to a layperson, of how acupuncture may help with fertility?	2 Points if yes and gives a reference, 1 point if yes but does not give a reference, 0 points if no
Does the website display data that clearly depicts its success rates?	1 Point if yes, 0 points if no
Is there a disclaimer that the treatment may not work?	1 Point if yes, 0 points if no
Are the risks made clear?	1 Point for 2 risks mentioned, 0 points if two are not mentioned
Are the side effects made clear?	1 Point for 2 side effects mentioned, 0 points if 2 are not mentioned
Are their reviews from previous patients?	1 Point if yes, 0 points if no
What education level do the acupuncturists have?	2 Points if any have a conventional medical degree, 1 point if they are therapists, 0 if no information provided
Does the website give any timing indication as to when the treatment should be started and for how long it should be continued?	1 Point if yes, 0 points if no
	Maximum points available = 12

This scoring system is not intended as an objective quantification; rather it is designed to provide a general indication of the extent to which the clinics provide sufficient information for the prospective patient. The reasoning behind the criteria in the scoring system is twofold. Firstly, information is a prerequisite for informed consent, without which the autonomy of the patient is jeopardised. Autonomy is a key requirement in medical ethics, and any diminution of this is therefore ethically problematic. Secondly, the medical profession is widely trusted by the public, and any lack of information or misinformation that violates this trust and influences a patient’s decision is considered unethical according to established norms of medical ethics. For example, Paragraph 71 of the GMC Good Medical Practice guidelines states:

*You must be honest and trustworthy when writing reports, and when completing or signing forms, reports and other documents. You must make sure that any documents you write or sign are not false or misleading.*

*a.You must take reasonable steps to check the information is correct.*

*b.You must not deliberately leave out relevant information* [16]

We consider this guidance to be equally pertinent in the setting of CAM fertility clinics’ website material. The above factors—autonomy and trust—are ethically crucial, and the scoring system used was intended to serve as a means to evaluate each website in respect of its ethical probity.^3^

## Results

Out of the initial 200 web search results, 48 of the CAM fertility clinics were identified as meeting the eligibility criteria.^4^

### Overall CIS

The average (modal) CIS score was 4, out of a possible 12. Scores ranged from 1 to 10.

### Explanation of acupuncture

Out of the 48 eligible CAM fertility clinic websites, 38 (79%) provided an explanation of what acupuncture is. The remaining 10 websites (21%) did not give an explanation.

### Description of procedure

Thirty-two (67%) of the websites provided a suitable description of how the acupuncture would be carried out. The remaining 16 websites (33%) did not give a description of the acupuncture procedure. It is also worth noting that none of the websites explained the techniques they would use to explore the medical profile of the patients.

### Fertility claims

Sixteen (33%) of the websites did not provide any reference to information on how acupuncture may help with infertility. Fifteen (31%) of the websites provided some information on how acupuncture may help with patient’s infertility; however, their information was not referenced to any journal, organisation, or clinical trial. Seventeen (35%) of the websites provided information on how acupuncture may help with patient’s infertility and also included at least one reference to the information source(s).

### Success rates

Forty-two (88%) of the websites did not provide any data on their success rates. Only 6 (13%) of the websites gave any information regarding successful outcomes. Of these 6, only 3 provided an actual rate; the remaining 3 did not refer to the period on which their claimed successes had occurred, meaning that a rate was not derivable. It is also worth noting that none of the websites referred to comparable rates from normal control subjects.

### Disclaimer

Ten (21%) of the websites provided a disclaimer which informed the patients that there is a lack of evidence regarding acupuncture’s efficacy and success. The remaining 38 websites (79%) did not display any disclaimer.

### Side effects and risks

Six (13%) of the websites gave a sufficient indication of the side effects that could occur from the acupuncture treatment. In terms of the risks of acupuncture, such as medical accidents, none of the websites made any reference to risks.

### Patient reviews

Fifteen (31%) of the websites contained reviews from patients. The remaining 33 websites (69%) either contained no patient reviews, or the reviews were not specific to the clinic itself.

### Education/training

Four (8%) of the websites declared that they employed conventional medical physicians. The remaining 44 (92%) of websites indicated that their therapists held acupuncture-specific (non-medical) qualifications.

### Timings and duration

Only 3 (6%) of the websites provided details of how long it may take for the acupuncture to be effective against infertility. The remaining 45 websites (94%) made no mention of the timeframe that a patient might expect.

### Additional aspects

The clinic websites were also analysed in respect of certain additional aspects.^5^ In summary: gender—29 (60%) of websites clearly indicated which gender they treated, of which the vast majority (90%) treated both men and women, with only 10% treating women; age—only 6 (13%) of websites indicated age restrictions, of which 2 (23%) restricted to women aged 40 years or below, and 4 (67%) treated women older than 40; and finally, very little clear information was presented by the websites on the following aspects: special IVF procedures, robotic micromachines and treatment of patients receiving chemotherapy.

## Discussion

This study describes the provision of acupuncture as a purported treatment for infertility by UK CAM fertility clinics, and evaluates the potential for misleading prospective patients. Two hundred CAM fertility clinics were initially identified, of which 48 met the eligibility criteria.

Assessment of the websites revealed that all the 48 cases had the potential to infringe on patient autonomy and put their well-being in jeopardy by giving them false medical information. This discovery is significant and carries substantial implications for regulatory agencies.

As far as we are aware, no previous studies that survey CAM fertility clinics, in respect of acupuncture or any other CAM therapies, have been published. Thus, the present study provides a unique perspective into a hitherto unexplored area in fertility medicine.

### Analysis of the results

The results of this study highlight several important ethical issues that could potentially impact infertile patients. Firstly, the fact that one-third of the clinics did not provide any information on the acupuncture procedure raises questions about informed consent. Patients have a right to know what treatments they are receiving and the risks and benefits associated with them. Without adequate information, patients may not fully understand the potential impact of the treatment, which could lead to dissatisfaction and mistrust in the healthcare provider.

Furthermore, the lack of evidence-based information on how acupuncture can help with infertility, as found on the majority of the websites, raises questions about the legitimacy of the treatment. It is essential for healthcare providers to provide evidence-based information to their patients, especially when dealing with such sensitive issues as infertility. The lack of references to any journal, organisation, or clinical trial can leave patients in the dark about the efficacy of the treatment, which could result in undue emotional and financial burden on them.

The absence of success rate data on most of the websites is also problematic. Patients should be provided with clear and accurate information on the success rates of treatments, as this can significantly impact their decision-making process. The lack of transparency in success rates can also lead to false hope, causing patients to undergo ineffective treatments for prolonged periods.

In addition, the lack of disclaimers on most of the websites is concerning, as it suggests that the clinics are promoting acupuncture as a proven treatment for infertility, which is not entirely accurate. Patients should be informed of the limitations of acupuncture and that there is a lack of evidence supporting its efficacy. Failure to do so can result in patients spending significant amounts of money on treatments that may not be effective.

The insufficient indication of side effects that could occur from the acupuncture treatment on most websites is also an ethical issue. Patients have the right to know the potential risks associated with any treatment they are receiving. Without such information, patients may not be able to give informed consent, leading to ethical issues such as deception.

Lastly, the fact that only a small percentage of clinics declared that they employed conventional medical doctors is also concerning. Infertility is a complex medical issue that requires a multidisciplinary approach. If a patient opts for alternative treatments such as acupuncture, this should be combined with conventional medical treatment.

### Strengths, limitations and future research

This study has several strengths, including its web-based content analysis approach, which allowed the researchers to quickly and easily access a large sample of CAM fertility clinics in the UK. The study’s scoring system also provides a useful tool for evaluating the quality of information provided on CAM fertility clinic websites. However, the study also has some limitations. For example, the study only analysed the information provided on the clinics’ websites and did not assess the quality of the treatment provided by the clinics. Therefore, the study’s findings cannot be generalized to the quality of the acupuncture treatment provided by the clinics.

Future research could clarify the degree to which patients are actually misled by the sort of problematic websites discovered in this study. Such research could also explore the events that happen beyond the websites, when potential patients actively engage with CAM fertility clinics. Ideally, the medical consultation process would be investigated. A better understanding of the modi operandi of the UK’s private CAM fertility clinics would provide valuable guidance for legislators to regulate appropriately, such that patients are no longer at risk of having their autonomy undermined by the sort of misleading information that, as this study demonstrates, presently dominates the websites of UK CAM fertility clinics.

### Implications of the findings

Where CAM clinics offer acupuncture for infertility, several ethical concerns and negative implications exist for patients. Firstly, patients may be vulnerable to false hope and deception. Acupuncture as a treatment for infertility is not supported by scientific evidence, and therefore, patients who invest significant amounts of money and time in such treatments may be exposed to a sense of false hope, leading to a waste of their resources and emotional distress.

Secondly, the promotion by CAM clinics of acupuncture for infertility may undermine the role of evidence-based medicine and the scientific method in healthcare. This may contribute to the general spread of medical misunderstandings and misperceptions among patients, who may choose to opt for infertility treatments that have not been scientifically proven to be effective, instead of seeking treatments that have been shown to work through rigorous clinical trials.

Thirdly, these practices can potentially result in misunderstanding and confusion among patients who may not be fully informed about conventional medical treatments or who may feel particularly pressured due to their fertility challenges. These individuals might find themselves facing high fees and undergoing additional treatments that might not be strictly necessary, leading to a financial burden and increased emotional stress.

Fourthly, neglecting to communicate the potential side effects and risks of acupuncture could inadvertently limit patients’ decision-making autonomy and might thereby expose them to unforeseen risks, due to a lack of full information. The websites imply that acupuncture is 100% ‘gentle’ and safe; in reality, pain or bleeding at the insertion site is a common side effect, and more serious accidents can occur. These include organ injury, infections, nerve damage, and miscarriage [17–20].

Finally, treating infertility with acupuncture will also have the negative consequence of causing patients to expend irrecoverable time in their efforts to conceive. As women age, their fertility decreases, and after the age of 35, the decline becomes more rapid. Therefore, delaying effective treatment for infertility can further reduce the chances of successful conception. Therefore, if patients invest significant amounts of time into being treated with an unproven CAM such as acupuncture, they may miss the opportunity to pursue more effective treatments, such as assisted reproductive technologies or other evidence-based medical interventions.

Thus, the promotion of acupuncture and other alternative therapies as treatments for infertility without adequate scientific evidence can lead to significant harm to patients, including not only financial and psychological distress but also a delay in seeking effective treatment, which could ultimately result in the loss of valuable time for patients, particularly women, as their proverbial biological clock ticks.

## Conclusions

This study provides a unique perspective into the use of acupuncture as a treatment for infertility in UK CAM fertility clinics. The results of this study highlight significant ethical issues that could potentially impact infertile patients, such as the lack of informed consent, evidence-based information, success rate data, disclaimers, and indication of side effects. The study’s scoring system provides a useful tool for evaluating the quality of information provided on CAM fertility clinic websites, and the findings carry substantial implications for regulatory agencies. The results indicate that problems exist in terms of the treatments and services offered by CAM fertility clinics, and the information they present to potential patients. The implications of the findings include the potential for raising false hopes, the possibility of vulnerable patients being misled (with concomitant issues of consent), and the expenditure of infertile patients’ valuable biological time that might otherwise have been better utilised pursuing proven medical treatments.

## Supplementary Information

Below is the link to the electronic supplementary material.

Supplementary file1 (DOCX 16 KB)

Supplementary file2 (DOCX 24 KB)

## Footnotes

1 Nevertheless, some of these otherwise bona fide ART clinics also offer fertility “add-ons”—namely, additional therapies and techniques designed to improve the chance of a live birth—and many of these add-ons lack a sound evidence-base (Day 2016). Examples of questionable add-ons include reproductive immunology treatments, assisted hatching, and time-lapse embryo imaging. The provision of these potentially lucrative but scientifically unverified ART enhancements has recently been criticised on scientific and ethical grounds in the medical press.

2 Established assisted conception treatments include in vitro fertilization (IVF), intracytoplasmic sperm injection (ICSI), intrauterine insemination (IUI), and gamete intrafallopian transfer (GIFT).

3 We consider that a CIS of 7 (from a possible 12) represented the minimal level that could be considered ethically acceptable. This threshold is of course subjective, and alternative levels are plausible. However, this imprecision does not detract from the overall conclusions of the study: where a clinic fails to fulfil even one of the information requirements, there is an ethical issue to be addressed.

4 See Supplemental file 1 for URLs of all 48 clinics analysed.

5 See Supplemental file 2 for a table of detailed findings.
