# Supplementary material for: Infertility clinics and acupuncture: a qualitative web-based study
Source: J Assist Reprod Genet. 2023 Aug 1;40(10):2367–73. doi: 10.1007/s10815-023-02898-6 (PMC10504127; doi:10.1007/s10815-023-02898-6)
Supplement: Supplementary file 1 — Supplementary file1 (DOCX 16 KB) [file 10815_2023_2898_MOESM1_ESM.docx]

**Supplemental File 1: Clinics meeting the inclusion criteria.**

| Clinic ID Number | Clinic Website Name |
| --- | --- |
| 1 | https://holistic-health.org.uk/ |
| 2 | <https://www.zitawestclinic.com/> |
| 3 | <https://www.londonacupuncture.co.uk/> |
| 4 | <https://heatonacupuncture.co.uk/> |
| 5 | <https://www.jesmondnaturalhealthandfertility.co.uk/> |
| 6 | <https://islingtonacupuncture.com/> |
| 7 | <https://www.acupuncturethatworks.co.uk/> |
| 8 | <https://www.chinamedic.co.uk/> |
| 9 | <https://hsfc.org.uk/> |
| 10 | <https://www.theforgeclinic.com/> |
| 11 | <https://www.leedsacupuncture.co.uk/index.html> |
| 12 | <https://orientalmed.ac.uk/icomclinic/> |
| 13 | <https://www.treatnorwich.co.uk/> |
| 14 | <http://www.unityfertility.co.uk/> |
| 15 | <https://healingspacehackney.co.uk/> |
| 16 | <https://shaftesburyclinic.com/> |
| 17 | <https://www.thehogarth.co.uk/> |
| 18 | <https://www.backandbodycareclinic.co.uk/> |
| 19 | <https://www.truehealthclinics.com/> |
| 20 | [https://www.conceive.org.uk](https://www.conceive.org.uk/) |
| 21 | <https://clinic.acumedic.com/> |
| 22 | <http://www.holistichealthhackney.co.uk/> |
| 23 | <https://www.acupuncture-works.co.uk/> |
| 24 | <https://almavalecentre.co.uk/> |
| 25 | <https://www.whitehartclinic.co.uk/> |
| 26 | <https://claphamcommonclinic.co.uk/> |
| 27 | <https://www.acupuncturehalifax.co.uk/> |
| 28 | <https://www.carmenta-life.co.uk/> |
| 29 | <https://www.wokingosteopaths.co.uk/> |
| 30 | <https://www.chelseanaturalhealth.co.uk/> |
| 31 | <https://hannahpearn.com/> |
| 32 | <https://www.taichiwellnesscentre.co.uk/> |
| 33 | <https://www.om-therapy.com/> |
| 34 | <https://liverpoolacupuncture.co.uk/> |
| 35 | <https://www.wetherbyholistichealth.co.uk/> |
| 36 | <http://btac.co.uk/> |
| 37 | <https://thesouthdownclinic.co.uk/> |
| 38 | <https://www.feelgoodbalham.co.uk/> |
| 39 | <https://www.chineseacupuncture-plymouth.co.uk/> |
| 40 | <https://www.positiveacupuncture.co.uk/> |
| 41 | <https://www.wellness-centre.co.uk/> |
| 42 | <http://www.thevalepractice.co.uk/> |
| 43 | <https://www.hilltopacupuncture.co.uk/> |
| 44 | <https://www.iffleyturnpractice.co.uk/> |
| 45 | <https://www.kenningtonosteopaths.co.uk/> |
| 46 | <https://actbrighton.org.uk/> |
| 47 | <https://www.woodsideclinic.co.uk/> |
| 48 | <https://www.victoriachiropractic.co.uk/index.html> |
