# Supplementary material for: Infertility clinics and acupuncture: a qualitative web-based study
Source: J Assist Reprod Genet. 2023 Aug 1;40(10):2367–73. doi: 10.1007/s10815-023-02898-6 (PMC10504127; doi:10.1007/s10815-023-02898-6)
Supplement: Supplementary file 2 — Supplementary file2 (DOCX 24 KB) [file 10815_2023_2898_MOESM2_ESM.docx]

**Supplemental File 2: Additional information.**

| ID  # | Clinic | Patient gender | Patient ages | Patients using chemo-therapy included? | Usage of robotic micro-machine? | Special procedures* applied? |
| --- | --- | --- | --- | --- | --- | --- |
| 1 | https://holistic-health.org.uk/ | Not stated | Not stated | Not stated | Not stated | Not stated |
| 2 | <https://www.zitawestclinic.com/> | Men and women | Up to 40+ (possibly higher but section for people 40+) | Not stated | Not stated | Not directly stated for acupuncture but they use acupuncture alongside IVF and cycle tracking etc |
| 3 | <https://www.londonacupuncture.co.uk/> | Men and women | Not stated | Not stated | Not stated | Not stated |
| 4 | <https://heatonacupuncture.co.uk/> | Men and women | Not stated | Not stated | No | No |
| 5 | <https://www.jesmondnaturalhealthandfertility.co.uk/> | Men and women | Not stated | Not stated | Not stated | Not stated |
| 6 | <https://islingtonacupuncture.com/> | Men and women | Not stated | Not stated | Not stated | Not stated |
| 7 | <https://www.acupuncturethatworks.co.uk/> | Women (also does not directly say they treat male but mentions a acu point for male infertility) | Not stated | Not stated | Not stated | Not stated |
| 8 | <https://www.chinamedic.co.uk/> | Men and women | Wide age range including people into their 40s | Not stated | Not stated | Not stated |
| 9 | <https://hsfc.org.uk/> | Women (possibly men too) | Not stated | Not stated | Not stated | yes |
| 10 | <https://www.theforgeclinic.com/> | Women (possibly men) | Wide variety (up to 40+) | Not stated | Not stated | Not stated |
| 11 | <https://www.leedsacupuncture.co.uk/index.html> | Men and women | Not stated | Not stated | Not stated | Not stated |
| 12 | <https://orientalmed.ac.uk/icomclinic/> | Adults (assume to be men and women) | Not stated | Not stated | Not stated | Not stated |
| 13 | <https://www.treatnorwich.co.uk/> | Men and women | Not stated | Not stated | Not stated | Not stated |
| 14 | <http://www.unityfertility.co.uk/> | Men and women | Not stated | Not stated | Not stated | Not stated |
| 15 | <https://healingspacehackney.co.uk/> | Men and women | Not stated | Not stated | Not stated | Not stated |
| 16 | <https://shaftesburyclinic.com/> | Women (possibly men – information unclear) | Not stated | Not stated | Not stated | Not stated |
| 17 | <https://www.thehogarth.co.uk/> | Men and women | Not stated | Not stated | Not stated | Not stated |
| 18 | <https://www.backandbodycareclinic.co.uk/> | Men and women | Not stated | Not stated | Not stated | Not stated |
| 19 | <https://www.truehealthclinics.com/> | Women (possibly men) | Not stated | Not stated | Electro-acupuncture | Not stated |
| 20 | [https://www.conceive.org.uk](https://www.conceive.org.uk/) | Men and women | Not stated | Not stated | Not stated | One of the acupuncturists can “also arrange western medical blood tests where necessary” |
| 21 | <https://clinic.acumedic.com/> | Men and women | Definitely women up to age 38 (could be older patients too but no reviews or anything to show for this), no data on male patient ages | Not stated | Not stated | Not stated |
| 22 | <http://www.holistichealthhackney.co.uk/> | Women (possibly men too) | Not stated | Not stated | No | Offers laboratory testing but more general like hormonal health, DNA profiling, neuro-transmitter metabolism so not specific for fertility but could possibly be used to flag up fertility related issues. |
| 23 | <https://www.acupuncture-works.co.uk/> | Men and women | Not stated | Not stated | Not stated | Not stated |
| 24 | <https://almavalecentre.co.uk/> | Men and women | Up to age 40 (from reviews) but may have treated older patients and not have a review | Not stated | No | No |
| 25 | <https://www.whitehartclinic.co.uk/> | Women (no mention of treatment for men) | Not stated | One person had acupuncture and had undergone chemo (does not say if it is related for fertility issues though) | Not stated | Not stated |
| 26 | <https://claphamcommonclinic.co.uk/> | See note** | n/a | Websites no longer exists/work | Websites no longer exists/work | Websites no longer exists/work |
| 27 | <https://www.acupuncturehalifax.co.uk/> | Does not directly state men, women or both (just conception issues/infertility) | Not stated | Not stated | No | No |
| 28 | <https://www.carmenta-life.co.uk/> | Men and women | Not stated | Not stated | Not stated | Yes |
| 29 | <https://www.wokingosteopaths.co.uk/> | Does not directly state men or women (boosts blood flow to reproductive organs but is not specific to male or female and also balances hormones but again not specific to male or female) | Not stated | Not stated | no | no |
| 30 | <https://www.chelseanaturalhealth.co.uk/> | Women (possibly men – information unclear) | Not stated | Not stated | no | no |
| 31 | <https://hannahpearn.com/> | Men and women | Not stated | Not stated | Not stated | no |
| 32 | <https://www.taichiwellnesscentre.co.uk/> | Men and women | Not stated | Not stated | no | no |
| 33 | <https://www.om-therapy.com/> | Women | Not stated | Not stated | No | No |
| 34 | <https://liverpoolacupuncture.co.uk/> | Men and women | Not stated | Not stated | No | No |
| 35 | <https://www.wetherbyholistichealth.co.uk/> | Women definitely, but works with couples (assuming both men and women but does not directly say this so only assumption) | Not stated | Not stated | No | No |
| 36 | <http://btac.co.uk/> | Not stated | Not stated | Not stated | No | No |
| 37 | <https://thesouthdownclinic.co.uk/> | Men and women | Ages through 3-92 (but not all applicable to fertility acupuncture) | Not stated | No | No |
| 38 | <https://www.feelgoodbalham.co.uk/> | Women (possibly men too as mentions couples acupuncture for infertility) | Not stated | Not stated | No | No |
| 39 | <https://www.chineseacupuncture-plymouth.co.uk/> | Men and women | Not stated | Not stated | No | No |
| 40 | <https://www.positiveacupuncture.co.uk/> | Women (no mention of men) | Women 40+ | Not stated | No | No |
| 41 | <https://www.wellness-centre.co.uk/> | Not stated (just mentions acupuncture for fertility) | Not stated | Not stated | No | No |
| 42 | <http://www.thevalepractice.co.uk/> | Men and women | Not stated | Not stated | Not stated | No |
| 43 | <https://www.hilltopacupuncture.co.uk/> | Does not state men or women (only for infertility) | Not stated | Not stated | Not stated | No |
| 44 | <https://www.iffleyturnpractice.co.uk/> | Not stated | Not stated | Not stated | No | No |
| 45 | <https://www.kenningtonosteopaths.co.uk/> | Men and women | Not stated | Not stated | No | No |
| 46 | <https://actbrighton.org.uk/> | Men and women | Not stated | Not stated | Not stated | No |
| 47 | <https://www.woodsideclinic.co.uk/> | Not stated | Not stated | Not stated | No | no |
| 48 | <https://www.victoriachiropractic.co.uk/index.html> | Not stated | Not stated | Not stated | No | No |

*Special procedures include oocyte denudation, fertilization rate, embryo vitrification and developmental competence.

**Website nonfunctional (date of access 29/06/2023); clinic may have ceased to operate.
